# Classification of adaptor proteins using recurrent neural networks and PSSM profiles

**DOI:** 10.1186/s12864-019-6335-4

**Published:** 2019-12-24

**Authors:** Nguyen Quoc Khanh Le, Quang H. Nguyen, Xuan Chen, Susanto Rahardja, Binh P. Nguyen

**Affiliations:** 1 Professional Master Program in Artificial Intelligence in Medicine, Taipei Medical University, Keelung Road, Da’an Distric, Taipei City 106, Taiwan (R.O.C.); 2 School of Information and Communication Technology, Hanoi University of Science and Technology, 1 Dai Co Viet, Hanoi 100000, Vietnam; 3 Beijing Genomics Institute, 21 Hongan 3rd Street, Shenzhen 518083, China; 4 School of Marine Science and Technology, Northwestern Polytechnical University, 127 West Youyi Road, Xi’an 710072, China; 5 School of Mathematics and Statistics, Victoria University of Wellington, Gate 7, Kelburn Parade, Wellington 6140, New Zealand

**Keywords:** Adaptor proteins, Prediction, Classification, Deep learning, RNN, GRU, PSSM

## Abstract

**Background:**

Adaptor proteins are carrier proteins that play a crucial role in signal transduction. They commonly consist of several modular domains, each having its own binding activity and operating by forming complexes with other intracellular-signaling molecules. Many studies determined that the adaptor proteins had been implicated in a variety of human diseases. Therefore, creating a precise model to predict the function of adaptor proteins is one of the vital tasks in bioinformatics and computational biology. Few computational biology studies have been conducted to predict the protein functions, and in most of those studies, position specific scoring matrix (PSSM) profiles had been used as the features to be fed into the neural networks. However, the neural networks could not reach the optimal result because the sequential information in PSSMs has been lost. This study proposes an innovative approach by incorporating recurrent neural networks (RNNs) and PSSM profiles to resolve this problem.

**Results:**

Compared to other state-of-the-art methods which had been applied successfully in other problems, our method achieves enhancement in all of the common measurement metrics. The area under the receiver operating characteristic curve (AUC) metric in prediction of adaptor proteins in the cross-validation and independent datasets are 0.893 and 0.853, respectively.

**Conclusions:**

This study opens a research path that can promote the use of RNNs and PSSM profiles in bioinformatics and computational biology. Our approach is reproducible by scientists that aim to improve the performance results of different protein function prediction problems. Our source code and datasets are available at https://github.com/ngphubinh/adaptors.

## Background

Protein function prediction is a technique that assigns biological or biochemical roles to proteins with regards to their genome sequences. The essential of understanding the protein function has drawn researchers’ attentions on enhancing the predictive performance of protein functions. Numerous solutions have been proposed in the past decades for this purpose. Two most effective solutions are finding strong feature sets and adopting powerful neural network models. Previous studies have revealed that using strong feature sets alone, for example, position specific scoring matrix (PSSM) [1], biochemical properties (AAindex) [2], and PseAAC [3], can achieve satisfactory prediction results. With the popularity of deep learning, many researchers in the field of bioinformatics attempted to apply the technique to protein function prediction. Some of the recent works like [4, 5] have demonstrated some successes. Motivated by these two observations, we intend to take the advantages of strong feature sets and deep neural network to further improve the performance by deriving a novel approach for protein function prediction. In this work, we put special focus to the prediction of adaptor protein, which is one of the most vital molecule function in signal transduction.

Signal transduction, so-called cell signaling, is the transmission from a cell’s outside to inside of molecular signals. Received signals must be transported viably into cells to guarantee a proper reaction. This progression is started by cell-surface receptors. One of the primary objectives of researchers who conduct their experiments on signal transduction is to decide the mechanisms that regulate cross-talk between signaling cascades and to decide the accomplishment of signaling. A rising class of proteins that much contributes to the signal transduction process are adaptor (or adapter) proteins. In adaptor proteins, there are numerious protein-binding modules linking protein-binding partners together. In addition, they are able to facilitate the signaling complexes creation [6]. They are vital in intermolecular interactions and play a role in the control of signal transduction started by commitment of surface receptors on all cell types.

In detail, adaptor proteins have been shown to be associated with a lot of human diseases. For instance, Gab adaptor proteins play an important role as therapeutic targets for hematologic disease [7]. XB130, a specific adaptor protein, plays an important role in cancer [8]. Likewise, Src-like adaptor proteins (SLAP-1 and SLAP-2) are important in the pathogenesis of osteoporosis, type I hypersensitivity, and numerous malignant diseases [9]. In [10], adaptor protein is also noted to be a therapeutic target in chronic kidney disease. Moreover, a review paper from [11] showed the association of adapter proteins with the regulation of heart diseases. Further, the involvement of adaptor protein complex 4 in hypersensitive cell death induced by avirulent bacteria has been shown in [12].

Given the significance of adaptor proteins to the functions and structures of signal transduction, elucidating the molecular mechanisms of adaptor proteins is therefore a very important research area which has recently gained rapid advancement. However, it is costly and time-consuming with these experimental techniques. Therefore, it is highly desired to develop automated prediction methods for quick and accurate identification of adaptor proteins.

PSSM is one of the most strong feature sets in biology to decode the evolutionary information of a protein sequence. Many computational studies have investigated the protein function prediction using PSSM profiles such as protein fold recognition [13], phosphoglycerylation prediction [14], succinylation prediction [15], and protein subcellular localization prediction [16]. However, among the existing approaches, none of them has found a solution to prevent the loss of amino acid sequence information in PSSM profiles. Here, to address this problem, we present an innovative approach via the use of a Recurrent Neural Network (RNN) architecture.

Standard neural network typically assumes independent relationship between input signals, but this is usually not the case in real world. Likewise, utilizing the co-relationship between genome sequences can help in protein function prediction.

We thus present a novel deep learning framework which utilizes RNNs and PSSM profiles to classify adaptor proteins. RNNs have been recently demonstrated to extract sequential information from sequences to predict various properties of protein sequences in several studies [17–19]. However, how to apply it on PSSM profiles to address the ordering information of them is still an open research question. The main contributions of this paper include (1) introducing a first sequence-based model for distinguishing adaptor proteins from general proteins, (2) proposing an efficient deep learning architecture constructed from RNNs and PSSM profiles for protein function prediction, (3) presenting a benchmark dataset and newly discovered data for adaptor proteins, and (4) providing valuable information to biologists and researchers for better understanding the adaptor protein structures.

## Results and discussion

### Experiment setup

Given an unknown sequence, the objective is to determine if the sequence is an adaptor protein and thus this can be treated as a supervised learning classification. As a representation, we defined adaptor protein as positive data with label “Positive”, and otherwise, non-adaptor protein as negative data with label “Negative”. We applied 5-fold cross-validation method in our training dataset with hyper-parameter optimization techniques. Finally, the independent dataset was used to evaluate the correctness as well as overfitting in our model.

Our proposed RNN model was implemented using PyTorch library with a Titan Xp GPU. We trained the RNN model from scratch using Adam optimizer for 30 epochs. The learning rate was fixed to 1×10^−4^ in the entire training process. Due to the significant imbalance in the sample numbers of adaptor proteins and non-adaptor proteins in the dataset, we adopted weighted binary cross-entropy loss in the training process. The weighting factors were the inverse class frequency.

Sensitivity, specificity, accuracy, and MCC (Matthew’s correlation coefficient) were used to measure the prediction performance. TP, FP, TN, FN are true positives, false positives, true negatives, and false negatives, respectively.

1 $$ \text{Sensitivity} = \frac{{TP}}{{TP + FN}}  $$

2 $$ \text{Specificity} = \frac{{TN}}{{TN + FP}}  $$

3 $$ \text{Accuracy} = \frac{{TP+TN}}{{TP + TN + FP + FN}}  $$

4 $$ \textrm{MCC} \,=\, \frac{{TP \times TN - FP \times FN}}{{\sqrt {(TP + FP)(TP + FN)(TN + FP)(TN + FN)} }}  $$

In addition, we also utilized Receiver Operating Characteristic (ROC) curves to examine the predictive performance of our model. In the ROC curve, the Area Under the Curve (AUC) metric is a floating point value ranging from 0 to 1 in which higher value represents better model. ROC curve and AUC are reliable metrics to compare the performance results among different models.

We first investigated the composition of amino acid in adaptor proteins and non-adaptor proteins to understand how we could better utilize the dataset for protein function prediction. We also studied how different hyper-parameters affected the performance of the RNN model. Besides, comparison between the proposed model and existing methods was based on the provided PSSM profiles.

### Comparison between adaptor proteins and non-adaptor proteins

**Fig. 1:**
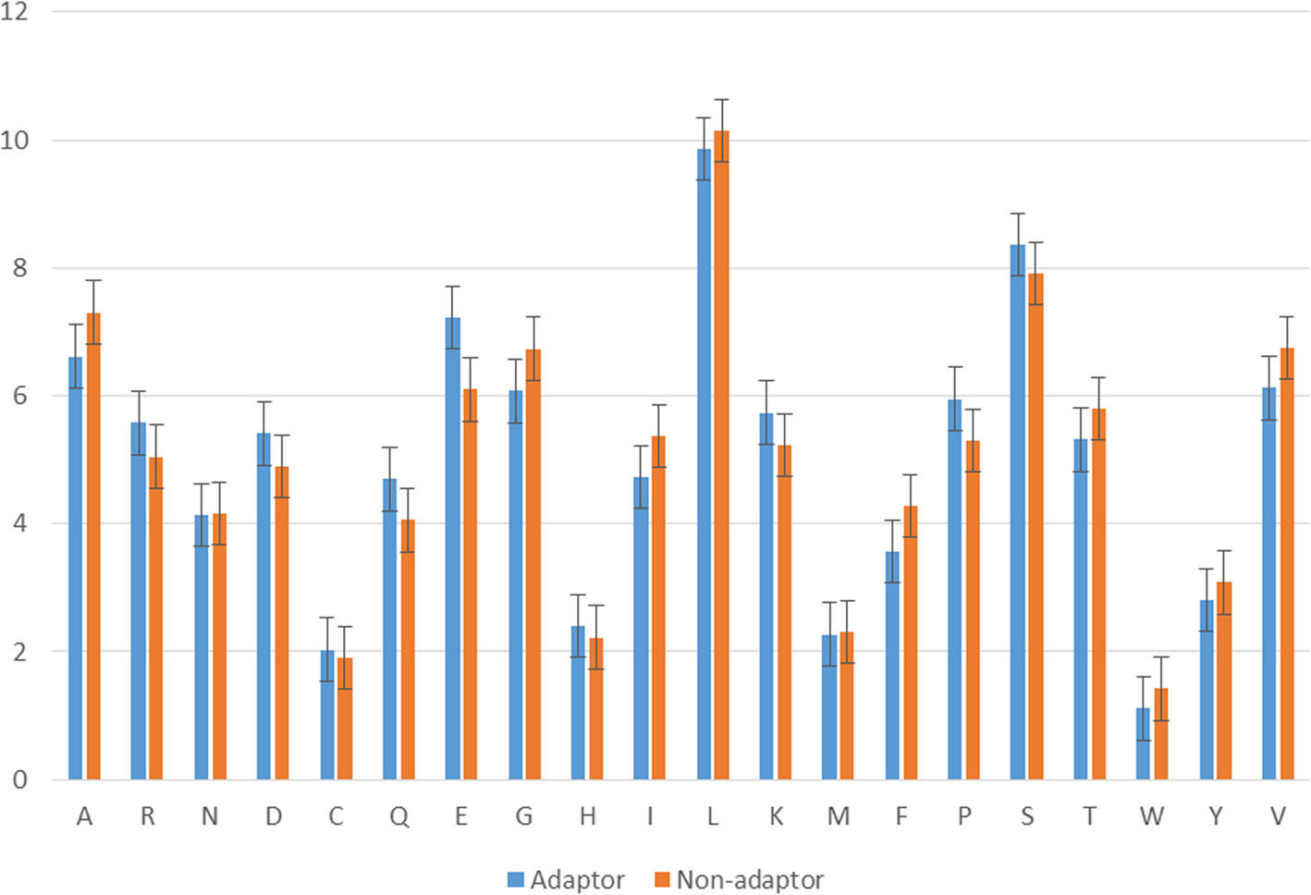
Different compositions of amino acid in adaptor proteins and non-adaptor proteins. *x*-axis represents 20 amino acids, *y*-axis represents the frequency (%) of each amino acid

We computed the amino acid frequency of adaptor and non-adaptor proteins in the whole dataset to analyze the differences between the two types. It can be seen from Fig. 1 that there are differences in amino acid composition surrounding adaptor and non-adaptor proteins. For example, the amino acid E, F, G, or V had higher variations to separate between two classes. The significant differences show that our model can distinguish adaptor proteins from general proteins according to some amino acid distributions.

### Study on selection of hyper-parameters

In this section, the selection of hyper-parameters is studied. Specifically, we have examined our model with different hyper-parameters, i.e., number of convolution filters, fully connected layer size, kernel size, and so on. We performed 5-fold cross-validation and varied the number of filters of the fully connected layer from 32 to 1,024 to find the optimal number. Our model has been selected based on the optimal performance results on validation dataset at a specific random seed value (i.e., random_seed = 7 in this study).

In our experiments, among different tested sizes, the fully connected layer size of 512 reached the maximum performance when discriminating the adaptor proteins in different validation settings. When testing our model in the independent dataset, the performance results were also consistent with the 5-fold cross-validation. It means that our model did not suffer from the over-fitting problem and can be applied in most of unseen data. A reason to explain this point is that we applied dropout, which is the regulation technique to prevent over-fitting in deep learning models.

The next important hyper-parameter that needs to be examined is the gated recurrent unit (GRU) hidden layer size. After several steps, we observed that the GRU with 256 hidden layer sizes was superior. Finally, these optimal parameters were used on our best model.

### Comparison between the current method and state-of-the-art techniques using pSSM profiles

After tuning up the hyper-parameters, we identified 512 filters and GRU size of 256 as the best performing architecture. We then used our optimized model to compare with the previous state-of-the-art methods. To use PSSM profiles, most recent techniques summed up all the same amino acids to produce a 400-dimensional vector and then fed to neural networks. A number of bioinformatics researchers have used this technique in their applications and obtained promising results [2, 5]. We also conducted experiments according to widely used machine learning algorithms including *k*-NN [20], the Random Forests (RF) [21] and the Support Vector Machines (SVM) [22]. Besides, we also compared our proposed method with a two-dimensional convolutional neural network (2-D CNN), which is a method treating PSSM profiles as images and successfully applied in sequence analysis [5].

Overall, the comparison between our proposed method and the other methods is shown in Table 1. Note that we used grid search cross-validation to find the optimal parameters of all algorithms. This ensures that our comparison is fair and reliable among these methods. The optimal results were: *k*=10 nearest neighbors in *k*-NN, 500 trees in RF, *c*=8 and *g*=0.5 in SVM, and 128 filters with each filter size of 3×3 in 2-D CNN. We easily observe that our RNN also exhibited the higher performance than the other techniques at the same level comparison. This was also supported by our preliminary work when testing with other classification algorithms including kernel dictionary learning [23–25] and an enhanced k-NN method [26]. It can be concluded that the sequential information of PSSM plays a vital role in predicting the adaptor protein as well as the other protein functions in general. Using 1D-CNN in our method helps to prevent the loss of sequential information compared to other embedding methods (e.g., 2D-CNN).

**Table 1:** Performance results of distinguishing adaptor proteins with different methods

Method	Cross Validation					Independent Test				
	Sensitivity	Specificity	Accuracy	AUC	MCC	Sensitivity	Specificity	Accuracy	AUC	MCC
*k*-NN	0.635	0.750	0.738	0.770	0.254	0.671	0.751	0.743	0.791	0.280
RF	0.185	**0.968**	**0.890**	0.837	0.214	0.290	0.923	0.860	0.838	0.216
SVM	0.397	0.934	0.881	0.818	0.332	0.426	**0.932**	**0.881**	0.806	0.353
CNN	0.532	0.875	0.841	0.774	0.328	0.548	0.873	0.841	0.783	0.339
RNN	**0.812**	0.751	0.757	**0.853**	**0.373**	**0.856**	0.798	0.804	**0.893**	**0.446**

(*k*-NN: *k*=10; RF: num_stimators=500; SVM: *c*=8.0, *g*=0.5; CNN: 128 filters; RNN: 512 filters)

Specifically, our sensitivity was significantly higher than that of the other methods. This is a very important point because our model aims to predict as much as adaptor proteins as possible. Via this high sensitivity, a large number of adaptor proteins could be discovered with a high accuracy. It provides a lot of information for biologists as well as researchers to understand and conduct their works on adaptor proteins.

**Fig. 2:**
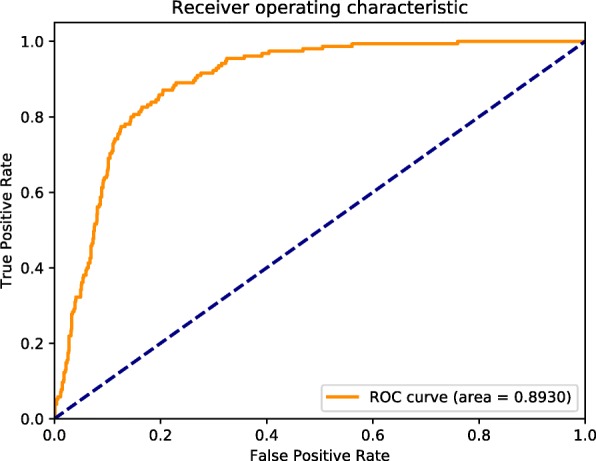
The receiver operating characteristic (ROC) curve of one fold in our experiments

The results in Table 1 are based on the default decision threshold value of each algorithm and this is not sufficiently significant. Hence, we show the ROC Curve and AUC to evaluate the performance results at different threshold levels. They are the most important evaluation metrics for checking the performance of most supervised learning classification. The ROC curve is plotted from True Positive Rate and False Positive Rate. As the value of AUC approaches to unity, the corresponding model is regarded to have shown optimal performance. As shown in Fig. 2, our model could predict the adaptor proteins with AUC of 0.893 and this is a significant level to show that our model performed well in this kind of dataset. It also determines that our results did not only perform well in a specific point but also at different levels. We can use this model to predict adaptor proteins with high performance and superior to the previous techniques (Table 1).

## Conclusions

In this study, we proposed an innovative method using RNN and PSSM profiles for distinguishing the adaptor proteins using sequence information only. It is also the first computational model that applies this combination to adaptor protein prediction. Via this method, we can conserve all the PSSM information in training process and to prevent the missing information as much as possible. The performance using 5-fold cross validation and independent testing dataset (including 245 adaptor proteins and 2,202 non-adaptor proteins) is evaluated. The proposed method could predict adaptor proteins with a 5-fold cross validation accuracy and MCC of 80.4% and 44.5%, respectively. To evaluate the correctness of our model, we applied an independent dataset testing and its accuracy and MCC achieved 75.7% and 37.3%, respectively. Our performance results are superior to the state-of-the-art methods in term of accuracy, MCC, as well as the other metrics.

This study discussed a powerful model for discovering new proteins that belong to adaptor proteins or not. This study opens a research path that can promote the use of RNN and PSSM profiles in bioinformatics and computational biology. Our approach is able to be reproduced by scientists that aim to improve the performance results of different protein function prediction problems.

Finally, physicochemical properties had been successfully used in a number of bioinformatics applications with high performance [27–29]. Therefore, it is possible to combine PSSM profiles and physicochemical proteins into a set of hybrid features. Subsequently, these hybrid features could be fed directly into our proposed architecture. We hope that the future studies will consider these hybrid features to help improving the performance results of protein function prediction.

## Methods

### Benchmark dataset

**Fig. 3:**
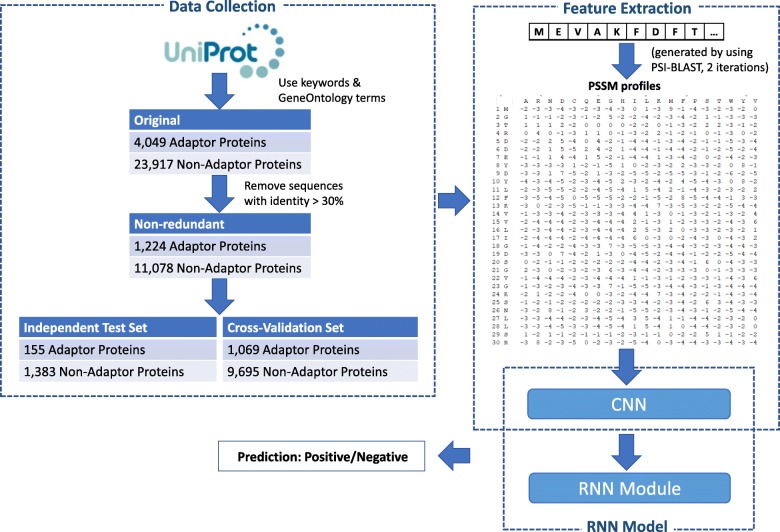
Flowchart of the study

Figure 3 illustrates the flowchart of the study. A detailed description on the construction of the benchmark dataset is provided as follows.

Because our study is the first computational study to classify adaptor proteins, therefore, we manually created a dataset from well-known protein data sources. We collected data from UniProt [30] and Gene Ontology (GO) [31], which provide high quality resources for research on gene products. We collected all the proteins from UniProt with GO molecular function annotations related to adaptor proteins. An important selection criteria is that we had to select the reviewed sequences, which means they had been published in scientific papers. Thus, the full query for collecting data was:

“keyword:“adaptor” OR goa:(“adaptor”)) AND reviewed:yes”

After this step, we received 4,049 adaptor proteins in all species.

We solved the proposed problem as a binary classification problem, thus we collected a set of general proteins as negative samples. Actually, our classifier aimed to classify between adaptor proteins and non-adaptor proteins. So we needed a real set of adaptors and non-adaptors to train the model. However, in practice, if we collect all non-adaptor proteins as negative data, the number of negative dataset will reach hundred thousands of data. This will result in serious data imbalance and affect the model’s performance. Therefore, in most of the related problems in bioinformatics, scientists can only select a subset of negative data and treat them as general proteins. In this study, we chose membrane protein, which is a general protein including a big enough number of sequences and functions. Briefly, we extracted all of the membrane proteins in UniProt and excluded the adaptor proteins. Similar to the previous step, only reviewed proteins were retained.

Subsequently, BLAST [32] was applied to all the collected data to remove redundant sequences with sequence identity level of more than 30%. This was an important step to prevent over-fitting in training model. The remaining sequences were regarded as valid for the benchmark dataset and were naturally divided into 1,224 adaptor proteins and 11,078 non-adaptor proteins. For fair comparison, we held up one-fifths of both the adaptor proteins and the non-adaptor proteins as the test set to evaluate model performance. The rest of the valid sequences were used as a cross-validation (Train-Val) set for model training. Table 2 lists the statistics of the benchmark dataset.

**Table 2:** Statistics of the benchmark dataset

	Original	Non-Redundant		
		Total	Train-Val	Test
Adaptor	4049	1224	1069	155
Non-Adaptor	23,917	11,078	9695	1383

### RNN model

**Fig. 4:**
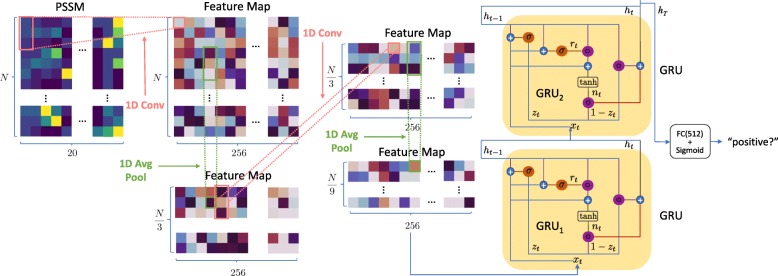
Architecture of the RNN model

In this study, we propose an RNN model for distinguishing adaptor proteins from non-adaptor proteins. An overview of the proposed RNN model is shown in Fig. 4. The RNN model takes PSSM profiles as inputs and extracts their features by several one dimensional (1-D) convolution layers and 1-D average pooling layers. The extracted features are then fed forward to gated recurrent units (GRUs), where the spatial context within the entire PSSM profile is explored and utilized for final prediction. The input sequence has a length of *N*. After going through two layers of 1-D CNN and 1-D Max-Pool, the length became *N*/9. Subsequently, this *N*/9 vector was fed into GRU, taking the output of GRU (256 features) to the input of the last vector for which the characteristic of the sequence was formed. Finally, our model took this output through a Fully Connected (FC) layer (512 nodes), and passed to a Sigmoid layer to produce a prediction probability value.

#### Preventing information missing by preserving ordering of pSSM profiles

A PSSM profile for a query protein is an *N*×20 matrix (*N* is the length of the query sequence), in which a score *P*_*ij*_ is assigned for the *j*^*th*^ amino acid in the *i*^*th*^ position of the query sequence with a large value and a small value indicating a highly conservative position and a weakly conservative position, respectively.

PSSM was first proposed by [1] and applied to various bioinformatics applications with promising improvements. The acquired protein sequences in the benchmark dataset are in FASTA format. From these FASTA sequences, we used PSI-BLAST [32] to generate PSSM profiles by searching them in the non-redundant (NR) database with two iterations.

Some studies attempted to predict the protein functions by summing up all of the same amino acids [2]. It helped to convert PSSM profiles with 20×*N* matrix to 20×20 matrix and all of the sequences had the same input length that can be easily used in supervised classification learning. However, important information could be lost since the ordering of PSSM profiles would be discarded. Therefore, an RNN architecture was presented to not only input PSSM profiles but also preserve the ordering. As the proposed RNN network accepts PSSM sequences with different lengths, we were thus able to well utilize their spatial context for better protein function prediction.

#### Feature extraction via CNN

The proposed RNN model first extracts convolutional features maps from PSSM profiles via an 1-D CNN. The CNN contains two 1-D convolution layers, each followed by a Rectified Linear Unit (ReLU) as non-linear activation, and two average pooling layers to reduce the dimension of the feature maps as well as enlarge the receptive field of the CNN network. The extracted feature maps are then fed forward to the RNN module for exploring the spatial relationship within the entire PSSM profile before final prediction.

#### Learning and classification using RNN

RNN is a neural network which had been shown to perform very well in various fields such as time series prediction [33], speech recognition [34], and language model [35]. Since RNN can memorize parts of sequential data, we used GRU which is an advanced architecture of RNN in this study.

After using the aforementioned CNN to create feature maps, we applied a multi-layer GRU to the extracted features. The standard RNN has a major drawback called the gradient vanishing problem, leading to that the network fails in memorizing information which is far away from the sequence and it makes predictions based on the most recent information only. Therefore, more powerful recurrent units, like GRU and Long Short-Term Memory (LSTM), were explored and introduced.

GRU is an advanced version of the standard RNN, in which the gradient vanishing problem is resolved by the introduction of an update gate and a reset gate for determining what information should be passed or discarded. GRU enables the possibility of long dependencies between the current input and far away information.

Basically, the structure of GRU is similar to LSTM. However, the fact that GRU requires less parameters than LSTM so it is more suitable for small datasets. This eases the training procedure and motivates us to adopt GRU as the basic unit in our RNN module. In the RNN module, a GRU layer consists of two gates:

(1) Update gate decides what information to throw away and what new information to add. To calculate the update gate *z*_*t*_, we used the following formula:

5 $$ z_{t} = \sigma \left(W_{iz} x_{t} + b_{iz} + W_{hz} h_{(t - 1)} + b_{hz} \right),  $$

where *t* is the time step, *σ* represents the sigmoid function, *W* represents weight, *x*_*t*_ represents the input at time *t*, *h*_(*t*−1)_ represents the hidden state of the previous layer at time *t*−1 or the initial hidden state at time 0, and *b* represents bias.

(2) Reset gate is applied in the model to determine how much past information to forget. The following formula is used:

6 $$ r_{t} = \sigma \left({W_{ir} x_{t} + b_{ir} + W_{hr} h_{(t - 1)} + b_{hr}} \right).  $$

Moreover, to save the past information from the reset gate, GRU uses a current memory content. It can be calculated using the following equation:

7 $$ n_{t} = \tanh \left({W_{in} x_{t} + b_{in} + r_{t} \circ \left({W_{hn} h_{(t - 1)} + b_{hn}} \right)} \right).  $$

Finally, the last step is final memory, to determine what to collect from the current memory content and the previous steps at the last step. To perform this step, GRU calculates vector *h*_*t*_ as follows:

8 $$ h_{t} = (1 - z_{t})\circ n_{t} + z_{t} \circ h_{(t - 1)}.  $$

The final output from the RNN module is then mapped to the prediction with a fully connected layer and the sigmoid function. The output from the RNN model is a scalar in [0,1] representing the probability that the PSSM profile belongs to the adaptor protein category (close to 1) or the non-adaptor protein category (close to 0).

## Data Availability

Our source code and datasets are available at https://github.com/ngphubinh/adaptors.

## Abbreviations

AUC: Area under the ROC curve
CNN: Convolutional neural network
GRU: Gated recurrent unit
LSTM: Long short-term memory
MCC: Matthew’s correlation coefficient
PSSM: Position specific scoring matrix
ReLU: Rectified linear unit
RF: Random forest
RNN: Recurrent neural network
ROC: Receiver operating characteristic
SVM: Support vector machine
